# H3.3S31 phosphorylation: linking transcription elongation to stimulation responses

**DOI:** 10.1038/s41392-020-00293-6

**Published:** 2020-08-29

**Authors:** Hong Wen, Xiaobing Shi

**Affiliations:** Center for Epigenetics, Van Andel Institute, Grand Rapids, MI 49503 USA

**Keywords:** Epigenetics, Epigenetics

Phosphorylation of histone proteins is involved in multiple cellular functions such as mitosis, DNA damage response, and transcriptional regulation. For example, phosphorylation of H3.3 Ser31 (H3.3S31ph) is known as a mitotic epigenetic modification.^1^ In a recent paper published on Nature, Armache et al. show that H3.3S31ph has an unexpected function in stimulation-induced transcription through crosstalk with H3K36me3, an epigenetic mark for transcription elongation.^2^ In eukaryotic cells, DNA is wrapped around histone octamers, which contain two copies of each of the core histones H2A, H2B, H3, and H4. Most histones, also known as canonical histones, are synthesized and deposited onto chromatin only during S phase when DNA is being replicated. There are also other histones, known as histone variants, that are expressed and synthesized in a cell cycle-independent manner. Incorporation of histone variants into nucleosomes provides a means to regulate the chromatin architecture in addition to other epigenetic mechanisms. Histone variants differ from canonical histones in just a few amino acids or large fractions of polypeptides. One of the histone variants, H3.3, has only four- or five-amino acid residue difference from canonical H3, namely H3.1 and H3.2, respectively. Three amino acids are located in the histone core domain important for binding to H3.3-specific histone chaperones. The only different residue at the N-terminus of the protein is at position 31, which is an Alanine in H3.1 and H3.2 but replaced by highly conserved Serine in H3.3. Phosphorylation of H3.3S31 was first reported 15 years ago,^1^ however, the function of this modification remains largely unknown.

By mass spectrometric analysis of purified histones from primary mouse bone marrow-derived macrophages (BMDMs), Armache et al. detected an increase of H3.3S31ph in lipopolysaccharide (LPS)-stimulated cells. Using a homemade H3.3S31ph specific antibody, they further corroborated that H3.3S31ph is a stimulation-associated histone modification in four additional cell types responding to different physiological stimuli. Chromatin immunoprecipitation followed by high-throughput sequencing (ChIP-seq) analysis revealed an enrichment of H3.3S31ph specifically in LPS stimulation-induced genes. The enrichment of H3.3S31ph in gene bodies was abolished or significantly reduced by treating the cells with P-TEFb inhibitor flavopiridol or TOP1 inhibitor camptothecin, both of which block the elongation of RNA polymerase II (Pol II), suggesting a direct link between H3.3S31ph and transcription elongation. Previous studies described that several kinases, such as IKKα, CHK1, and aurora B, are able to phosphorylate H3.3S31. A series of genetic perturbation using short hairpin RNA (shRNA) and pharmacological inhibition experiments suggested that IKKα is likely responsible for the deposition of co-transcriptional H3.3S31ph at inflammatory genes.

The distribution pattern of H3.3S31ph in gene body is reminiscent of that of another transcription elongation-associated histone modification, H3K36me3. H3K36me3 is mediated by a single histone methyltransferase, SETD2. Given this link between H3.3S31ph and H3K36me3 as well as their physical proximity on the H3.3 tail, Armache et al. hypothesized that H3.3S31ph may provide stimulation-induced genes with the capacity for augmented transcription, in part through the stimulation of H3K36me3. Indeed, the crystal structure of the human SETD2 catalytic domain bound to a H3.3 peptide bearing S31ph and related biochemical data supported this hypothesis. Two basic residues of SETD2, K1600, and K1673, provide positive charge recognizing the phosphorylated H3.3S31, thus promoting the engagement of H3.3K36 at the catalytic site for methylation. Consistent with the structural observation, SETD2 showed higher catalytic activity on the phosphomimic H3.3S31E-containing nucleosomes over unmodified nucleosomes; however, the stimulatory effect was lost by mutating any of the two basic lysine residues to glutamine (K1600E or K1673E). Together, these data suggest that IKKα stimulates target gene expression by H3.3S31ph-medaited augmentation of SETD2 activity.

Previously, we and others identified ZMYND11 as an H3.3K36me3 specific reader that functions as a transcriptional corepressor and tumor suppressor.^3^ In addition to K36me3, unmodified S31 also contributes to the binding. Structural and biochemical data suggested that a phosphate at Ser31 impedes ZMYND11 binding to H3.3K36me3. Consistent with these in vitro data, inflammatory genes tend to have pre-existing H3.3K36me3 with coincident ZMYND11 occupancy in resting BMDMs; whereas upon stimulation, ZMYND11 is rejected from these genes. This is likely a consequence of IKKα-mediated H3.3S31ph, as ZMYND11 is retained at inflammatory genes in cells treated with an IKK inhibitor before LPS stimulation. Finally, to directly define the function of H3.3S31ph in inflammatory gene induction, Armache et al. generated H3f3a H3f3b double knockout (DKO) RAW264.7 macrophage cell lines using CRISPR-Cas9 and rescued the DKO cells with the wild-type, H3.3S31A (loss of function) and H3.3S31E (gain of function, phosphomimic) transgenes. Overall, LPS-induced gene expression in H3.3 DKO macrophages was increased by wild-type H3.3 or H3.3S31E transduction, but not by the H3.3S31A mutant, suggesting an essential role of H3.3S31ph in promoting rapid stimulation-induced transcription in macrophage-like cell lines.

**Fig. 1:**
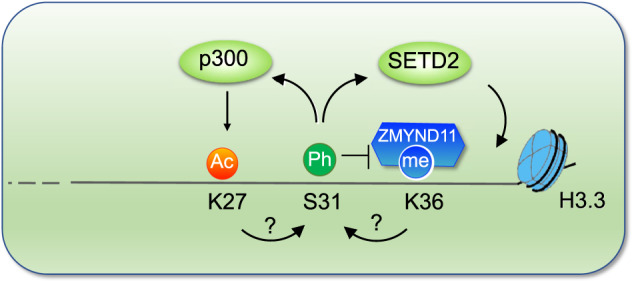
Crosstalk between variant-specific phosphorylation on H3.3S31 and other modifications. H3.3S31ph promotes SETD2-dependent trimethylation on H3K36 and inhibits binding of H3K36me3 by the transcription corepressor ZMYND11, thus enabling rapid and robust gene expression. H3.3S31ph can also enhance the enzymatic activity of the histone acetyltransferase p300 to promote transcription by H3K27 acetylation. Ac acetylation. Me methylation. Ph phosphorylation

Despite that H3.3S31ph was first reported as a mitotic epigenetic modification 15 years ago,^1^ its function in interphase remained unknown. Studies from two laboratories recently showed that H3.3S31ph stimulates the catalytic activity of histone acetyltransferase p300, thus maintaining enhancers at an open, acetylated chromatin state permissive to the embryonic development program.^4,5^ In the current study, Armache et al.^2^ discovered a novel role of H3.3S31ph in transcription elongation. It not only augments the catalytic activity of SETD2, but also ejects negative regulator ZMYND11. Thus, together with H3.3K36me3, H3.3S31ph provides an epigenetic signature in gene bodies promoting rapid and high-level expression of stimulation-induced genes (Fig. 1). The region of histone H3 tail between K27 to K36 has become a research focus in the chromatin field, in part because of frequent mutations of histone H3K27, G34, and K36 in various pediatric tumors. Interestingly, many of these mutations occur in H3.3-encoding genes. Given the importance of the crosstalk between H3.3S31ph and modifications at neighboring residues in transcription regulation, it would be interesting to determine in future studies the role of H3.3S31ph in the pediatric tumors bearing the deadly histone mutations.
